# Molecular Dynamics Simulation and Experimental Study of the Mechanical and Tribological Properties of GNS-COOH/PEEK/PTFE Composites

**DOI:** 10.3390/polym16182572

**Published:** 2024-09-11

**Authors:** Zhen Dong, Henan Tang, Bin Yang, Shijie Wang, Yunlong Li, Lin Liu

**Affiliations:** School of Mechanical Engineering, Shenyang University of Technology, Shenyang 110870, Chinali_yunlong3390@163.com (Y.L.);

**Keywords:** PTFE, PEEK, graphene, molecular dynamics, experiment

## Abstract

Molecular dynamics (MD) simulations were first employed to achieve the optimal sintering temperature of carboxyl-functionalized graphene (GNS-COOH)-modified polyether ether ketone (PEEK)/polytetrafluoroethylene (PTFE) composites. A model of GNS-COOH/PEEK/PTFE composites was constructed to simulate the effects of different sintering temperatures on the mechanical and tribological properties, as well as their underlying atomic mechanisms. Samples of PTFE composites were prepared and characterized through experimental methods. Results revealed that the sintering temperature significantly affects the intermolecular forces, mechanical properties, and tribological characteristics of the composites. The agglomeration of the PEEK/PTFE composite matrix was effectively mitigated by introducing GNS-COOH. When the sintering temperature was controlled at 360 °C, the compressive strength of GNS-COOH/PEEK/PTFE composites was improved compared to GNS/PEEK/PTFE composites, albeit with a slight reduction in wear resistance. This study provides a theoretical reference for the preparation process and performance evaluation of new materials.

## 1. Introduction

Polytetrafluoroethylene (PTFE) is a general-purpose engineering plastic with a variety of excellent properties, including self-lubrication, high- and low-temperature resistance, and stable chemical inertness [1,2]. However, PTFE needs to be modified due to its unsatisfactory wear resistance. The polyether ether ketone (PEEK) has been proven and can substantially enhance the wear resistance and deformation resistance of PTFE-based composites [3]. Bijwe [4] and Burris [5] investigated the mechanism of improving the tribological properties of PEEK-enhanced PTFE composites. Carbon nanomaterials, represented by GNS, are widely recognized as promising nanofillers for the development of nanocomposites [6,7]. Xie et al. [8] used graphene (GNS) filled with PTFE to study the preparation process and its properties of GNS/PTFE composites. Functionalization modification on the GNS structure can further improve the agglomeration phenomenon of GNS/PTFE composites [9,10,11,12]. In our previous study, we found that 1%GNS/10%PEEK/PTFE composites have more excellent tribological properties compared to 10%PEEK/PTFE, but the blending process of GNS is often accompanied by the agglomeration effect. In this paper, hydroxyl-functionalized graphene (GNS-COOH)-filled PEEK/PTFE composites were used in order to solve the GNS agglomeration phenomenon and to investigate the mechanical and frictional properties of GNS-COOH-filled PEEK/PTFE composites.

In the preparation of PTFE composites, the sintering temperature is an important factor in the preparation process. Ye et al. [13] studied the effect of sintering temperature on the mechanical properties of PTFE, and the results showed that the sintering temperature has a large impact on the properties of PTFE materials. The degree of sintering will lead to abnormal changes in the average molecular weight of the blanks, resulting in a change in the mechanical properties. At this stage, the research on the preparation and properties of PTFE-based composites mainly adopts experimental methods, which have a long test period and consume a lot of materials. The molecular force field theory and molecular dynamics (MD) simulation technology provide more technical means for the research and preparation process of new materials. Compared with experimental research, MD simulation shows the advantages in efficiency and can explain the intrinsic mechanism from a microscopic point of view. Numerous MD simulations of GNS, PTFE, and PEEK have been conducted to investigate the microscopic mechanisms underlying the experimental behavior of these composites [14,15,16,17,18].

Based on the MD simulation analysis method, the molecular model of GNS-COOH/PEEK/PTFE composites was constructed. Different sintering temperatures in the preparation of GNS-COOH/PEEK/PTFE composites were varied by simulation to investigate the effect of the sintering temperature on the mechanical and tribological properties. The influence law of the sintering temperature on composites was investigated to obtain a better sintering temperature. Then, the samples of GNS-COOH/PEEK/PTFE and GNS/PEEK/PTFE composites were prepared and experimentally characterized the mechanical and tribological properties of the specimens. This study establishes a research paradigm by using MD simulations to determine material preparation parameters, thereby guiding the preparation and testing of experimental samples. It provides a valuable reference for the development and fabrication of new materials.

## 2. Modeling of Composites

**Figure 1:**
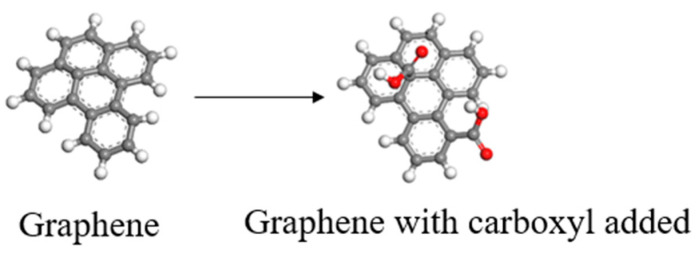
Carboxyl-functionalized graphene.

**Figure 2:**
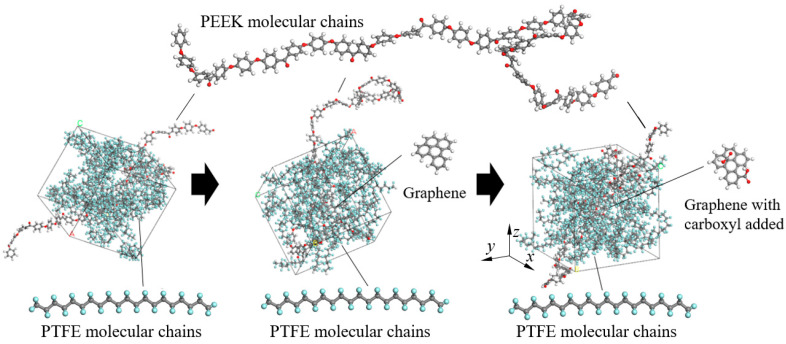
Construction of the molecular amorphous hybrid model.

GNS was firstly constructed using Material Studio 8.0 software and then carboxyl-functionalized to obtain GNS-COOH with an oxidation degree of 24.24%, as shown in Figure 1. Based on the Monte Carlo “random number” calculation method [19], 10 repetitive units of PTFE chains and 10 repetitive units of PEEK chains were filled into a cubic lattice with a size of 3 × 3 × 3 nm^3^ and periodic boundary condition using the *Homopolymer* function in the *Build* module. The density of the model was set to 2.1 g/cm^3^. The convergence accuracy of the force field in the calculation was set to 0.001 kal/mol/Å, and the energy field was set to 10^−5^ kcal/mol [20]. The mass ratio of PTFE to PEEK was 9:1, and the mass fraction of GNS was 1%. The amorphous composite model (1%GNS-COOH/10%PEEK/PTFE) was constructed, as shown in Figure 2.

The model was optimized using the *Forcite* module to obtain the minimum energy configuration of the model. The geometry optimization was performed using the most rapid descent method and conjugate gradient method. Then, the global dynamics optimization was performed by the *Dynamics* command for the NVT and NPT systems, respectively. The optimization temperature was set to 298 K, and the optimization time was 1000 ps. The COMPASS force field was employed, which is suitable for the construction and calculation of polymer composites [21]. The time step was set at 1 fs, and the pressure was set at 101 KPa in all the MD simulations. A cutoff radius of 12.5 Å was applied in all the simulations. Andersen was chosen for the temperature controller and Berendsen for the pressure controller.

The optimized model was heated up from 25 °C (298 K) to 340 °C, 350 °C, 360 °C, 370 °C, and 380 °C with 200 ps NVT kinetics optimization to simulate different sintering temperatures during the preparation process. The molecular amorphous models of 1% GNS-COOH/10% PEEK/PTFE at different temperatures were obtained.

## 3. Result and Discussion

### 3.1. Mechanical Properties of MD Simulations

The optimized model with different sintering temperatures was used to conduct the tensile process using the constant strain method. The constant strain rate was set to 0.1%. Then, the mechanical property parameters of the 1%GNS-COOH/10% PEEK/PTFE composites such as the Young’s modulus, the bulk modulus, and the shear modulus were obtained and analyzed. According to the definition of the Vieri stress, the stress components in each direction of the composites can be calculated and Young’s modulus can be calculated using the following equation:

*E*_i_ *= σ*_i_*/ε*_i_; *σ*_i_ *= C*_ij_*·ε*_i_; *ε*_i_ *= S*_ij_*·σ* (1)

where *σ*_i_ is the stress, *ε*_i_ is the strain, *C*_ij_ is the stiffness matrix, and *S*_ij_ is the flexibility matrix.

**Figure 3:**
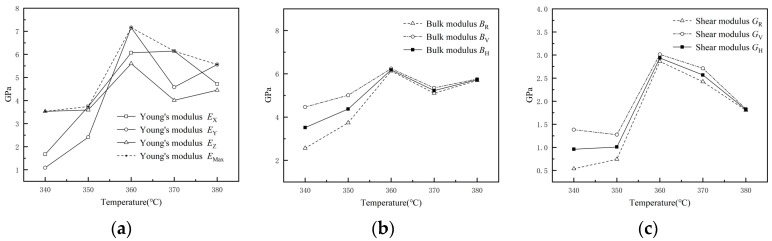
Mechanical property parameters of MD simulations. (**a**) Young’s modulus; (**b**) Bulk modulus; (**c**) Shear modulus.

The volumetric modulus *B* and shear modulus *G* of the composites can be calculated according to the Voigt–Reuss–Hill theory, and the actual volumetric modulus *B*_H_ and shear modulus *G*_H_ of the composites can be estimated, in which *E*_X_, *E*_Y_, and *E*_Z_ denote Young’s modulus in the three directions of x, y, and z, respectively; *E*_Max_ is the maximum Young’s modulus in the three directions; *B*_R_ and *B*_V_ are the Reuss volumetric modulus and Voigt volumetric modulus, respectively; *G*_R_ and *G*_V_ are the Reuss shear modulus and Voigt shear modulus, respectively; and *G*_H_ and *B*_H_ are the Hill volume modulus and Hill volume modulus, respectively, and their values are the average values of the Reuss modulus and Voigt modulus. The mechanical properties of the 1%GNS-COOH/10%PEEK/PTFE composites are listed in Table 1, and the variation in mechanical properties with the temperature trend is shown in Figure 3.

As can be seen from Table 1 and Figure 3, the sintering temperature obviously affected the mechanical properties of the composites. A reasonable explanation for this observation is that the change in temperature will affect the intermolecular forces and the crystallization of the base material PTFE, which makes the composites in the performance of different mechanical properties [22]. When the sintering temperature is 360 °C, the Young’s modulus of the composites reaches a maximum value of 7.16 Gpa, the average value of bulk modulus reaches a maximum value of 6.18 Gpa, and the average value of shear modulus reaches a maximum value of 2.94 Gpa. It indicates that the composites in this sintering temperature show stronger flexural, tensile, deformation, and shear resistance properties.

### 3.2. Tribological Properties of MD Simulations

#### 3.2.1. Modeling

**Figure 4:**
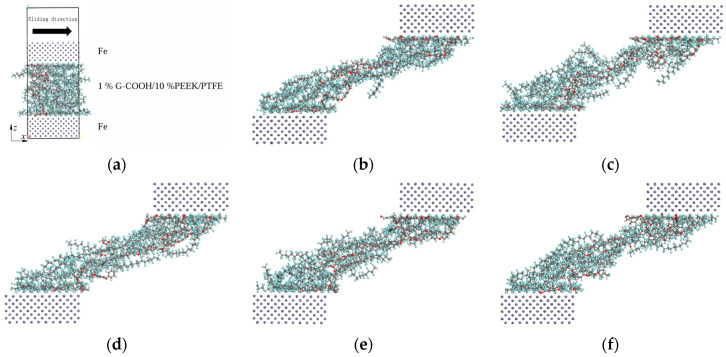
Friction simulation model (**a**) and snapshots of the models at the sintering temperature of 340 °C (**b**), 350 °C (**c**), 360 °C (**d**), 370 °C (**e**), and 380 °C (**f**).

The shear models were built using the *Forcite* module to simulate the friction process. Firstly, the Fe atoms are retrieved and expanded using the *Supercell* command, and the shear sub-model of the Fe layer was built as the friction partner. The *Build layers* command was used to construct the molecular models of the frictional pairs, and the intermediate layer was the 1%GNS-COOH/10%PEEK/PTFE model, as shown in Figure 4a. The Fe layers were fixed at first, and the geometry optimization of the intermediate layer was carried out using the most rapid descent method and the conjugate gradient method. The energy convergence tolerance was set to 10^−5^ kcal/mol, and the force convergence tolerance was set to 0.001 kcal/mol/Å. Then, the Fe layers were given a relative sliding velocity of 0.1 Å/ps for the friction simulations under the 500 ps NVT ensembles. The simulation time step was set to 1 fs and the initial temperature to 298 K. The friction simulations at each sintering temperature were accomplished using the *Confined Shear* command, and the snapshots of models were intercepted at 300 frames, as shown in Figure 4b–f.

In Figure 4, it is obvious that the molecular chains of the composites will cause slippage and fracture phenomena in the friction process with the changes in the strength of the intermolecular forces, which in turn affects the tribological performance of the composites [23].

#### 3.2.2. Friction Coefficient and Wear Rate

After the friction simulation, the average friction coefficient and wear rate of each model were calculated separately. The friction coefficient was determined by the following equation:

*μ* = *F*_f_*/F*_n_; *F*_f_ = *F*_O_ + *τA*_C_(*L*) (2)

where *F*_n_ is the normal force, which refers to the component force along the normal direction of the external force applied to the composites. *F*_f_ is the friction force, which refers to the force that hinders the tendency of the composites to move relative to each other during the friction process. *F*_O_ and *τ* denote the load-independent Derjaguin offset and effective shear strength, respectively, and *A*_C_(*L*) denotes the area of frictional contact under the action of the load *L*.

The wear rate of friction was determined by the following equation:

*AR = N*_leave_/*N*_total_ (3)

where *N*_leave_ and *N*_total_ denote the number of atoms leaving the matrix during the friction process and the total number of atoms in the original matrix, respectively [24].

**Figure 5:**
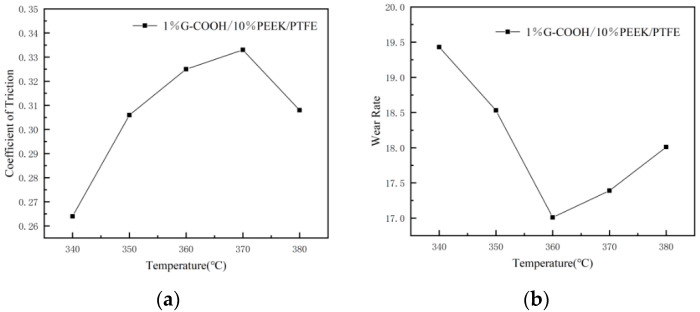
Trend of friction coefficient (**a**) and wear rates (**b**).

The average friction coefficient and wear rate of the composites at different sintering temperatures are shown in Table 2. The trend of friction coefficient of the composites is shown in Figure 5a and the trend of wear rate is shown in Figure 5b.

From Table 2 and Figure 5, the highest coefficient of friction of the composites was achieved when the sintering temperature at 370 °C. When the sintering temperature was at 360 °C, the wear rate of the composites was the lowest (17.01%).

#### 3.2.3. Radial Distribution Functions and Relative Atomic Concentrations

**Figure 6:**
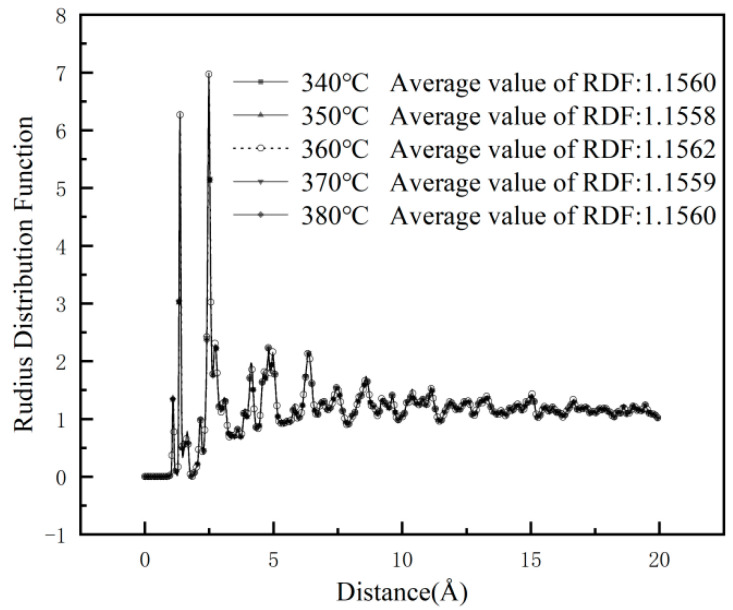
RDF at different sintering temperatures.

To investigate the effect of sintering temperature on the mechanical and tribological properties of 1%GNS-COOH/10%PEEK/PTFE composites and its microscopic mechanism, the relative atomic concentration and radial distribution function (RDF) along the z-direction of the composites were introduced. The RDF is an indicator parameter to analyze the interaction between atoms and characterize the micro-distribution between the polymers, which reflects the interaction of molecular chains at the friction interfaces. The mechanism influencing the mechanical and tribological properties of the composites can be explained by characterizing the strength between the molecular chains. The RDF and its mean values of the models at each sintering temperature are shown in Figure 6.

In Figure 6, the distance *r* corresponding to the peaks of the RDF is analyzed. The type of intermolecular interactions can be derived: peaks at *r* < 3.1 Å indicate chemical and hydrogen bonding interactions, peaks at 3.1 Å < *r* < 5.0 Å indicate strong van der Waals interactions, and peaks at *r* > 5.0 Å indicate weak van der Waals interactions [25]. In the distance range of 1~3.1 Å, two peaks appeared in the composites when r = 1.37 Å and 2.49 Å, respectively. It indicates that the type of intermolecular forces in the composites is mainly dominated by chemical bonding and hydrogen bonding interactions, followed by van der Waals forces. When the sintering temperature is 360 °C, the average value of the RDF of the model was the largest, indicating that the model has the strongest intermolecular forces, and the molecular chains show stronger cohesion during friction simulation.

**Figure 7:**
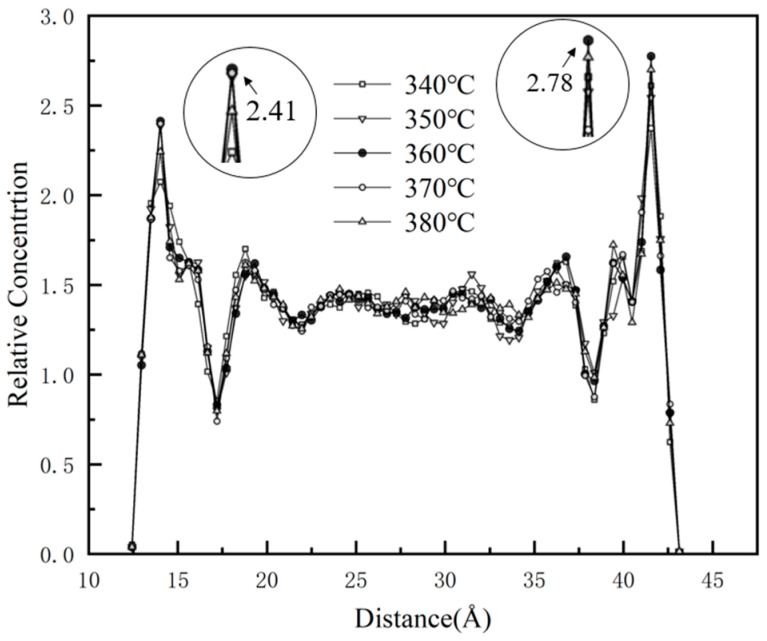
Relative atomic concentration distribution along the *z* direction.

The relative atomic concentration distribution of 1%GNS-COOH/10%PEEK/PTFE composites along the *z*-direction is shown in Figure 7. The peaks appear at 14 Å and 41 Å in the *z*-direction during the friction process at all sintering temperatures, with both peaks achieving the maximum value at 360 °C sintering temperature. The maximum value of the peak at 41 Å increases by 14.1% compared to the minimum peak value at 14 Å and 14.7% to the minimum peak value at 41 Å. Combined with the RDF data, it indicates that the composites have stronger adsorption between the molecular chains when the sintering temperature reaches 360 °C. The molecules between GNS-COOH and PEEK/PTFE were subjected to hydrogen bonding and van der Waals forces to form a more compact structure, which is macroscopically manifested in the enhancement of the mechanical and tribological properties.

#### 3.2.4. Mean Square Displacement

**Figure 8:**
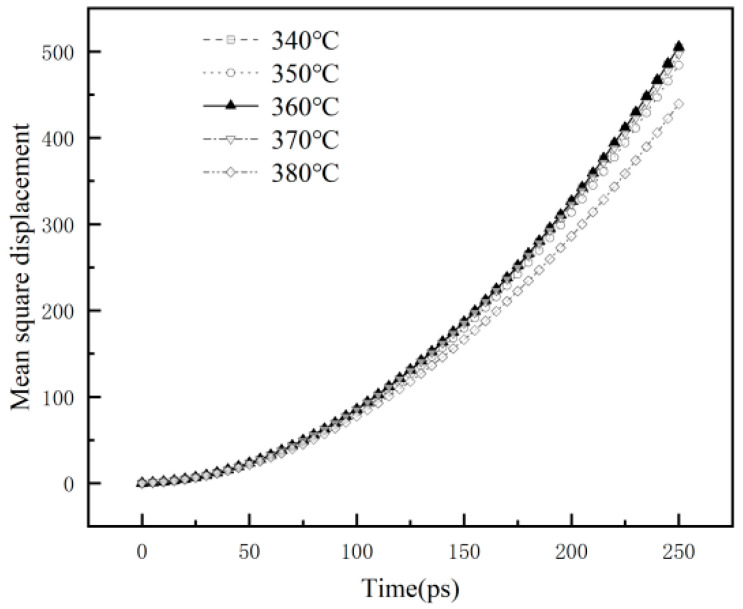
Mean square displacement of the molecular chains.

To further investigate the microscopic mechanism of the sintering temperature on the properties of composites. The mean square displacements (MSD) of the molecular chains in the 1%GNS-COOH/10%PEEK/PTFE composites were calculated, as shown in Figure 8.

The average mean square displacement (MSD) values of the composites were 1.73 Å^2^, 1.66 Å^2^, 1.74 Å^2^, 1.72 Å^2^, and 1.53 Å^2^ at sintering temperatures of 340 °C, 350 °C, 360 °C, 370 °C, and 380 °C, respectively. The maximum average value of the MSD appeared at the sintering temperature of 360 °C, indicating that the molecules of the composite matrix have stronger interfacial interactions with each other and limit the relative mobility. Hence, the 1%GNS-COOH/10%PEEK/PTFE composites in this state have better integrity and stability, making the molecules more difficult to transfer from the matrix to the friction interface, which in turn improves the mechanical and tribological properties of the composites in the sintering temperature of 360 °C [26,27,28].

Comprehensive simulation studies revealed that the composites with a sintering temperature of 360 °C had optimal mechanical and tribological properties. To obtain first-hand experimental data to guide industrial production, 1%GNS-COOH/10%PEEK/PTFE composites were prepared. The tests on mechanical and tribological properties were carried out.

### 3.3. Experimental Preparation and Testing

#### 3.3.1. Materials and Preparation

PTFE powder: Daikin Fluorochemical (China) Co., Ltd., Changshu, China with an average particle size of 40 μm; PEEK powder: Jilin Zhongyan Polymer Materials Co., Ltd, Changchun, China with an average particle size of 24 μm; GNS: Suzhou Tanfeng Graphene Technology Co. Ltd, Suzhou, China with multilayered graphene powder and an average sheet diameter of 25 μm.

GNS was added to an aqueous hydrogen peroxide solution (with a 10%volume fraction of hydrogen peroxide) for ultrasonic cleaning and then added to a KH550 silane coupling agent-acetone solution for ultrasonic dispersion treatment, followed by preparation of GNS-COOH by the chemical reagent method.

PEEK powder was dried at a temperature of 150 °C for 3 h. PTFE powder was screened with a 10-mesh sieve. PEEK and PTFE powder were pre-mixed according to the mass fraction of 1:9 and put into a high-speed mixer for full mixing as substrate. Then, 1% GNS-COOH was added to the substrate powder and mixed under the same conditions. Cold pressing and sintering techniques were used for the preparation of composites

The sintering process of 1%GNS-COOH/10%PEEK/PTFE composites is as follows: the substrate was heated to 320 °C from 25 °C, with a heating rate of 50 °C/hour, and kept at this temperature for 30 min. Then, the substrate was heated to the predetermined sintering temperature (360 °C) with a heating rate of 30 °C/hour and the temperature was kept constant for 3 h. Furthermore, the substrate was cooled down to 320 °C with a cooling rate of 30 °C/hour and kept at this temperature for 30 min. Finally, the substrate was cooled from 320 °C to 250 °C with a cooling rate of 70 °C/hour and powered off to naturally reduce to room temperature. The composites containing GNS (denoted as 1%GNS/10%PEEK/PTFE) were prepared simultaneously for experimental control, and the sintering process was the same as above.

#### 3.3.2. Experimental Methods

The tensile test was conducted following GB/T 1040.2-2022 [29] and the compression test was conducted following GB/T 1041-2008 [30]. The tensile and compressive properties of the composites were tested using the AG-X vertical electronic universal testing machine (Shimadzu, Kyoto, Japan). The tensile test samples were prepared according to the 5A dimensional standard specified with a length of 80 mm, a nominal axis cross-sectional diameter of 6 mm, and a gauge length of 25 mm. The tensile speed was 50 mm/min. The size of the compression specimen was 10 × 10 × 4 mm^3^, and the compression speed was 1 mm/min. The friction test was conducted using a reciprocating friction and abrasion tester under 200 N load, 2 Hz frequency, and room temperature. The test conditions were dry friction without lubrication and the test time of each sample was 0.5 h. The size of the friction test sample was 30 × 7 × 6 mm^3^, the reciprocating stroke was 10 mm, and the speed was 0.04 m/s. The friction vice was QT450 with the hardness of HBW170. The mass of the worn samples was weighed by precision balance before and after the test. The morphology of the samples after the friction test was observed and analyzed by SEM using Zeiss Sigma 300 (Carl Zeiss AG, Oberkochen, Germany).

#### 3.3.3. SEM Elemental Analysis

**Figure 9:**
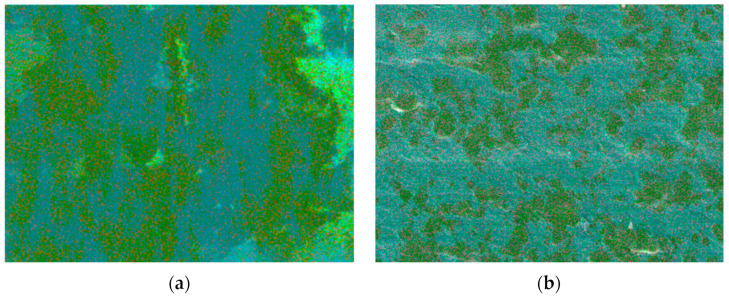
Elemental layered electron images (500×). (**a**) 1%GNS/10%PEEK/PTFE; (**b**) 1%GNS-COOH/10%PEEK/PTFE.

The prepared sample surfaces were firstly analyzed by SEM elemental layering scanning, and the elemental layered electron image micro-morphology of 1%GNS-COOH/10%PEEK/PTFE and 1%GNS/10%PEEK/PTFE composites were obtained, respectively, as shown in Figure 9.

**Figure 10:**
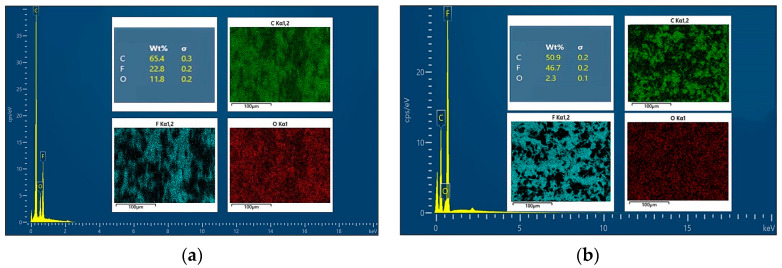
Total spectrum of elemental distribution maps. (**a**) 1%GNS/10%PEEK/PTFE; (**b**) 1%GNS-COOH/10%PEEK/PTFE.

In Figure 9, the elemental distribution of 1%GNS-COOH/10%PEEK/PTFE composites was more uniformly dispersed than that of 1%GNS/10%PEEK/PTFE, and the spatial arrangement of the components was more regular. The continuity between the elements of 1% GNS/10% PEEK/PTFE composites was more powerful, and the spatial arrangement of the components was more centralized, but elemental distribution uniformity was poor. To further investigate the effect of GNS-COOH on the distribution of matrix components, the total spectrum of elemental distribution maps of the composites were extracted and spectra, as shown in Figure 10.

In Figure 10, the element C content of 1%GNS-COOH/10%PEEK/PTFE composites decreased by 20.8% compared to 1%GNS/10%PEEK/PTFE. The mass percentage of element F of 1%GNS-COOH/10%PEEK/PTFE composites increased by 51.2% compared to 1%GNS/10%PEEK/PTFE composites, the mass percentage of element O was reduced by 80.5%. The element distribution of the composites introduced by GNS was more inhomogeneous and continuous than that of the composites introduced by GNS-COOH. The element distribution of the composites containing GNS-COOH was more clearly expressed and dispersed, and the spacing of the components was more obvious, which indicated that the linkage between the molecular chains was weaker. Combined with the electronic images of elemental distribution in Figure 9, it can be visualized that the composites of 1%GNS-COOH/10%PEEK/PTFE have significantly improved component agglomeration compared to the 1%GNS/10%PEEK/PTFE.

#### 3.3.4. Mechanical Properties Study

Based on the tensile and compression experiments, the mechanical property parameters of 1%GNS/10%PEEK/PTFE and 1%GNS-COOH/10%PEEK/PTFE composites such as tensile strength, compressive strength, and modulus of elasticity were obtained and listed in Table 3.

In Table 3, the tensile strength of 1%GNS-COOH/10%PEEK/PTFE composites decreased by 27.1%, compressive strength increased by 37.2%, and elongation at break decreased by 42.2% compared to 1%GNS/10%PEEK/PTFE. The compressive strength of 1%GNS-COOH/10%PEEK/PTFE composites was slightly improved, while the tensile strength was decreased compared to 1%GNS/10%PEEK/PTFE composites. Combined with the description in Section 3.3.3, it is reasonable to speculate that the GNS-COOH makes the spatial arrangement of the components in the composites more dispersed and reduces the stress concentration. It makes the 1%GNS-COOH/10%PEEK/PTFE composites superior to the 1%GNS/10%PEEK/PTFE composites in compressive resistance. However, the dispersion of the components destroys the connectivity and interaction between the molecular chains and decreases the tensile properties compared with 1%GNS/10%PEEK/PTFE composites.

#### 3.3.5. Tribological Properties Study

The parameters of tribological properties of 1%GNS/10%PEEK/PTFE and 1%GNS-COOH/10%PEEK/PTFE composites such as abrasion losses, friction coefficient, and wear rate were obtained and listed in Table 4.

**Figure 11:**
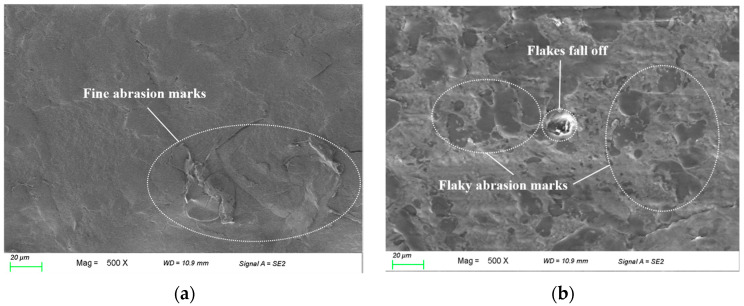
SEM micro-morphology images of the samples. (**a**) 1%GNS/10%PEEK/PTFE; (**b**) 1%GNS-COOH/10%PEEK/PTFE.

In Table 4, the abrasion losses of 1%GNS-COOH/10%PEEK/PTFE composites increased by 37.8% compared to 1%GNS/10%PEEK/PTFE composites. The coefficient of friction had a slight change while the wear rate increased by 38.4% compared to 1%GNS/10%PEEK/PTFE composites. The wear resistance of 1%GNS-COOH/10%PEEK/PTFE composites decreased compared to 1%GNS/10%PEEK/PTFE composites. The micro-morphology of the samples after wear at 500× magnification was further observed and shown in Figure 11.

As can be seen in Figure 11, the surface morphology of 1%GNS/10%PEEK/PTFE composites was smoother than that of 1%GNS-COOH/10%PEEK/PTFE composites, which were dominated by scale-like abrasions with no obvious flaky shedding and scratches. This is due to the GNS and PEEK components effectively inhibiting the fracture and slip of the PTFE macromolecular chains. It can be observed in Figure 11 that the 1%GNS/10%PEEK/PTFE composites have a strong connection between the components and show excellent wear resistance. In contrast, the micro-morphology of the 1%GNS-COOH/10%PEEK/PTFE composites was rougher and the wear marks were obvious, with unevenly distributed flake wear marks and pearly groove wear marks along the friction direction, and there is also flake shedding. A reasonable explanation for these observations is that the components of the 1%GNS/10%PEEK/PTFE composites had different wear resistance, and the addition of a carboxyl functional group disrupts the interactions between the molecular chains of the composites and decreases the intermolecular force. Hence, the macroscopic manifestation of the composites after friction test microscopic morphology appeared in a large number of uneven color flake abrasion marks. This conclusion corresponds to the tribological performance of composites.

## 4. Conclusions

(1) The MD simulation shows that the mean value of RDF of the 1%GNS-COOH/10%PEEK/PTFE composites reaches the maximum at the sintering temperature of 360 °C, while the mean values of relative atomic concentration and MSD also reach the maximum. The intermolecular forces (including hydrogen bonding and van der Waals forces) of the composites are the strongest at this temperature, which makes better mechanical properties and wear resistance of the composites;

(2) Samples of 1%GNS-COOH/10%PEEK/PTFE and 1%GNS/10%PEEK/PTFE composites were prepared for mechanical and friction test. 1%GNS-COOH/10%PEEK/PTFE composites were more dispersed and had clearer boundaries of the components, the tensile strength decreased by 27.1% and compressive strength increased by 37.2% compared to 1%GNS/10%PEEK/PTFE composites;

(3) 1%GNS-COOH/10%PEEK/PTFE composites vs. 1%GNS/10%PEEK/PTFE composites have similar friction coefficients, with a 38.4% reduction in wear resistance.

## Figures and Tables

**Table 1:** Mechanical property parameters of MD simulations (GPa).

Sintering Temperature	*E*_X_	*E*_Y_	*E*_Z_	*E*_Max_	*B*_R_	*B*_V_	*B*_H_	*G*_R_	*G*_V_	*G*_H_
340 °C	1.67	1.09	3.53	3.53	2.57	4.47	3.52	0.54	1.38	0.96
350 °C	3.74	2.41	3.59	3.74	3.74	5.01	4.37	0.74	1.28	1.01
360 °C	6.07	7.16	5.60	7.16	6.13	6.24	6.18	2.86	3.01	2.94
370 °C	6.14	4.58	4.01	6.14	5.10	5.35	5.23	2.42	2.71	2.57
380 °C	4.72	5.56	4.45	5.56	5.69	5.76	5.73	1.80	1.83	1.82

**Table 2:** Friction coefficient and wear rate.

Sintering Temperature	Frictional Coefficient	Wear Rate (%)
340 °C	0.264	19.43%
350 °C	0.306	18.53%
360 °C	0.325	17.01%
370 °C	0.333	17.39%
380 °C	0.308	18.01%

**Table 3:** Mechanical property parameters of composites in experiments.

Composite Materials	Tensile Strength (MPa)	Elongation at Break (%)	Compressive Strength (MPa)
1%GNS/10%PEEK/PTFE	21.1	216%	104.2
1%GNS-COOH/10%PEEK/PTFE	15.4	174%	165.9

**Table 4:** Tribological properties parameters of composites in experiments.

Types of Composite Materials	Abrasion Losses (mg)	Coefficient of Friction	Wear Rate
1%GNS/10%PEEK/PTFE	2.8	0.122	0.067%
1%GNS-COOH/10%PEEK/PTFE	4.5	0.126	0.108%

## Data Availability

The original contributions presented in the study are included in the article; further inquiries can be directed to the corresponding author/s.
